# Blockade of Myd88 signaling by a novel MyD88 inhibitor prevents colitis-associated colorectal cancer development by impairing myeloid-derived suppressor cells

**DOI:** 10.1007/s10637-022-01218-6

**Published:** 2022-01-28

**Authors:** Lu Wang, Dan Hu, Bin Xie, Lin Xie

**Affiliations:** 1 Institute of Organ Transplantation, Tongji Hospital, Tongji Medical College, Huazhong University of Science and Technology; Key Laboratory of Organ Transplantation, Ministry of Education, China; NHC Key Laboratory of Organ Transplantation, China; Key Laboratory of Organ Transplantation, Chinese Academy of Medical Sciences, Wuhan, China; 2 Department of Neurology, Renmin Hospital of Wuhan University, Wuhan, China

**Keywords:** MyD88 signaling, Myeloid-derived suppressor cells, Colitis-associated colorectal cancer, MyD88 inhibitor

## Abstract

*Background.* In cancer, myeloid-derived suppressor cells (MDSCs) are known to escape the host immune system by developing a highly suppressive environment. However, little is known about the molecular mechanism behind MDSC-mediated tumor cell evasion of the immune system. Toll-like receptor (TLR) signaling elicited in the tumor microenvironment has the potential to induce MDSC differentiations in different organs. Therefore, MDSC elimination by blocking the action of myeloid differentiation factor 88 (MyD88), which is a key adaptor-signaling molecule that affects TLR activity, seems to be an ideal tumor immunotherapy. Previous studies have proven that blocking MyD88 signaling with a novel MyD88 inhibitor (TJ-M2010-5, synthesized by Zhou’s group) completely prevented colitis-associated colorectal cancer (CAC) development in mice. *Methods.* In the present study, we investigated the impact of the novel MyD88 inhibitor on the number, phenotype, and function of MDSC in the mice model of CAC. *Results.* We showed that CAC growth inhibition was involved in diminished MDSC generation, expansion, and suppressive function and that MDSC-mediated immune escape was dependent on MyD88 signaling pathway activation. MyD88 inhibitor treatment decreased the accumulation of CD11b^+^Gr1^+^ MDSCs in mice with CAC, thereby reducing cytokine (GM-CSF, G-CSF, IL-1β, IL-6 and TGF-β) secretion associated with MDSC accumulation, and reducing the expression of molecules (iNOS, Arg-1 and IDO) associated with the suppressive capacity of MDSCs. In addition, MyD88 inhibitor treatment reduced the differentiation of MDSCs from myeloid cells and the suppressive capacity of MDSCs on the proliferation of activated CD4^+^ T cells in vitro. *Conclusion.* MDSCs are primary cellular targets of a novel MyD88 inhibitor during CAC development. Our findings prove that MyD88 signaling is involved in the regulation of the immunosuppressive functions of MDSCs. The novel MyD88 inhibitor TJ-M2010-5 is a new and effective agent that modulates MyD88 signaling to overcome MDSC suppressive functions, enabling the development of successful antitumor immunotherapy.

## Introduction

As tumors may escape the host immune system by developing a highly suppressive environment, an increasing number of studies have focused on tumor immune evasion [1]. Tumor immune evasion is mediated by factors released from a tumor or by immunosuppressive cells infiltrating the tumor microenvironment, such as myeloid-derived suppressor cells (MDSCs), natural killer (NK) cells, macrophages, dendritic cells (DCs), eosinophils and regulatory T cells (Tregs) [2]. Tilting the balance from an immunosuppressive environment to an immune-active environment may be an effective in tumor therapy [3, 4].

An increased count of MDSCs has been found in the peripheral blood (PB), bone marrow (BM), lymph nodes, and tumor sites of patients and experimental animals with tumors. MDSCs have been confirmed to suppress host antitumor immunity and promote tumor progression [5–7]. Therefore, eliminating MDSCs may be an ideal tumor immunotherapy strategy [8, 9]. Recently, several treatment approaches have been suggested to overcome MDSC-induced immunosuppression [10]. The key point of these strategies is to either decrease the number of expanding MDSCs or to attenuate their immunosuppressive activity in tumor-bearing host.

Although the mechanism behind MDSC generation, expansion, and suppressive function is not completely known, recent studies have suggested that MDSC accumulation is mediated by tumor-associated mediators, including granulocyte macrophage-colony stimulating factor (GM-CSF) [11], interleukin (IL)-1β [12], IL-6 [13], vascular endothelial growth factor (VEGF) [14] and transforming growth factor-β (TGF-β) [15]. Furthermore, the main immunosuppressive mechanism driven by MSDCs involves inhibition of T cell responses [16], suppression of NKT-mediated lysis [17], polarization macrophages toward the type 2 form [18], limitation of the availability of mature DCs [19] and promotion of Treg expansion [6] in the tumor-bearing host.

Sustained Toll-like receptor (TLR) activation is known to be associated with persistent inflammatory cytokine production and tissue damage. Moreover, TLR signaling elicited in the inflammatory or tumor microenvironment has the potential to induce MDSCs in different organs. TLR2/6 heterodimers expand and recruit MDSCs to the skin to suppress T cell expansion [20, 21]. Lipopolysaccharide (LPS) promotes MDSC development in a TLR4-dependent manner to suppress T cell function in the lungs [22]. TLR9 blockade inhibits the suppressive activity of MDSCs on T cell proliferation in tumor-bearing mice [23]. Therefore, activation of the TLR signaling pathway may play a critical role in MDSC-mediated tumor immune evasion.

Myeloid differentiation factor 88 (MyD88) is a key adaptor-signaling molecule for all TLRs except TLR3 [24]. MDSCs have been proven to inhibit inflammation in the lungs as they are TLR4/MyD88-induced cells that can influence T cell responses [22, 25]. MyD88^−/−^ MDSCs fail to suppress the tumor antigen-specific T cell immune response [26]. Moreover, our previous study proved that the administration of a novel MyD88 inhibitor, TJ-M2010-5 (a patented small-molecule compound synthesized by Dr. Zhou’s group), which has been proven to be effective in suppressing the innate immune response in many animal models of diseases (such as hepatocellular carcinoma [27], acute liver injury [28], tracheal, cardiac and skin transplantation [29, 30], graft-versus-host disease [31] and myocardial ischemia reperfusion injury [32]), may completely prevent the development of colitis-associated colorectal cancer (CAC) in mice by controlling inflammation and carcinogenesis [33]. Therefore, we investigated the impact of the novel MyD88 inhibitor on the number, phenotype, and function of MDSCs in mice with CAC. Here, we focus on the underlying molecular mechanisms of the MyD88 inhibitor targeting MDSCs and provide novel insights into therapeutic strategies targeting MDSCs in tumor-bearing hosts and cancer patients.

## Methods

### Animals

Female BalB/c mice (6 weeks old) were obtained from Weitonglihua Company (Beijing, China). All animals were housed in a specific pathogen-free facility. The experimental protocol was approved by the Animal Care and Research Committee of Huazhong University of Science and Technology.

### Colitis-associated colorectal cancer model

CAC in mice was induced as previously described. To induce CAC, each mouse was injected intraperitoneally (i.p.) with 10 mg/kg azoxymethane (AOM, Sigma-Aldrich Chemical, Germany). Seven days later, the mice began to receive three cycles of 2.5% dextran sodium sulfate (DSS, MP Biomedicals, USA) in drinking water for one week and two weeks of regular drinking water throughout a 10-week observation period. A group of age- and gender-matched healthy control mice received vehicle (sterile water) injection and plain drinking water. Samples (colon, spleen, PB, and BM) were harvested at weeks 5,7, 8 and 10 (W5, W7, W8 and W10, respectively).

### MyD88 inhibitor treatment

All mice were randomly divided into the normal control (NC), CAC model (CAC), and MyD88 inhibitor-treated groups (I, n = 4–5 per group). The mice in the MyD88 inhibitor group were treated with 50 mg/kg TJ-M2010-5 (i.p.) daily beginning two days before the first DSS administration throughout the 10-week-observation period. The mice in the control group were injected with the same volume of sterile water.

### Evaluation of colorectal tumors

The entire colon of each mouse was opened longitudinally, and the number of tumors in each colon was counted. The paraffin-embedded colon tissue Sects. (4 µm) were stained with hematoxylin–eosin (HE).

## Preparation of single-cell suspensions

### Lamina propria mononuclear cells (LPMCs)

LPMCs were isolated from colonic tissues using a lamina Ppropria (LP) dissociation kit, according to the manufacturer's instructions (Miltenyi Biotec, Germany, in Supplementary Methods).

### BM cells

Femurs of mice were aseptically removed and debrided of surrounding muscle tissue. BM cells were flushed from the femur using phosphate buffer saline (PBS).

### Splenocytes

The spleens of the mice were excised and sliced into small pieces. The excised pieces were pressed through a strainer, and the harvested cells were washed with PBS.

### PBMCs

PBMCs were isolated from freshly obtained whole blood of mice using density gradient separation (TBD sciences, China, in Supplementary Methods).

### Antibodies (Abs)

The Abs used in this study are shown in the Supplementary Methods.

### Flow cytometry analysis

Quantitative flow cytometric analyses were performed using standard procedures. For analysis of intracellular cytokine production, cells were permeabilized for 20 min at 4 ℃. Data acquisition was performed using a FACS Celesta flow cytometer, and they were analyzed by FlowJo software (Version 10.0).

### Immunohistochemistry (IHC) and immunofluorescence (IF)

The paraffin-embedded colon tissue Sects. (4 µm) were incubated with Abs. The bound Abs were detected sequentially with biotin-conjugated secondary antibody and streptavidin-HRP and visualized with a DAB kit (Beyotime Biotec, China) for IHC. For IF, Alexa Fluor 488 anti-rabbit or Cy3 anti-rat Abs (Servicebio, China) were used. The slides were mounted with DAPI (Servicebio, China) and examined under a fluorescence microscope.

### Cell sorting with magnetic beads

CD3^−^ splenocytes, CD11b^−^ BM cells, CD11b^+^Gr-1^+^ MDSCs and CD4^+^ splenic T cells were purified by magnetic bead negative/positive selection according to the manufacturers’ instructions (Miltenyi Biotec., Germany).

### Western blot analysis

Colon tissue samples were homogenized and separated by SDS-PAGE for Western blot analysis. Cell lysates from sorted CD11b^+^Gr-1^+^ splenocytes were prepared and diluted using a sample preparation kit (Protein Simple) for the automated capillary Western blot system, WES System (Protein Simple, in the Supplementary Methods).

### RAW264.7 cell culture and stimulation

The RAW 264.7 cell line was obtained from the China Center for Type Culture Collection (CCTCC #GDC143, Wuhan, Hubei). Frozen aliquots were used in the experiments within six months of culture period, after the first thawing of the cells. The cell line has been tested and authenticated. Cells were cultured in RPMI 1640 medium (Gibco, USA). After stimulation with 100 ng/mL LPS (Sigma-Aldrich Co., Germany) for four hours after 40 μM TJ-M2010-5 one-hour pretreatment; cells and the supernatant in individual wells were harvested for mRNA extraction and ELISA, respectively.

### Quantitative real-time polymerase chain reaction (RT-qPCR)S

Total RNA was extracted from mouse colon tissues or RAW264.7 cells using TRIzol reagent (Invitrogen, USA). cDNA was synthesized with a Thermo_Fisher kit (USA). RT-qPCR was performed using the Hieff qPCR SYBR Green Master Mix (Yeasen Biotech., China) and a StepOne System (Life Technologies, USA). Relative fold changes were determined using the ΔΔCT calculation method. Values were normalized to the internal control β-actin. The sequences of the primers are given in the Supplementary Methods.

### ELISA

The concentrations of GM-CSF, IFN-γ, IL-1β, IL-6, and TGF-β1 in the supernatant were analyzed by ELISA according to the ELISA kit manufacturer’s instructions (eBioscience, USA).

### In vitro MDSC differentiation from immature myeloid cells

CD11b^−^ BM cells were flushed from the femur using PBS and grown in RPMI medium supplemented with 10% heat-inactivated FBS (Yeasen Biotech., China), 1 mM sodium-pyruvate (Meilunbio, China), and 50 μM β-mercaptoethanol (Solarbio, China). Cultures were supplemented with 10 ng/mL GM-CSF (PeproTech, USA) and 1 μg/mL LPS and incubated at 37 ℃ for 8 days. At the end of the culture period, the population of CD11b^+^Gr-1^+^ MDSCs that had differentiated from immature myeloid cells was identified by flow cytometry.

### In vitro suppressive activity of MDSCs on CD4^+^ T cell proliferation

CD11b^+^Gr-1^+^ MDSCs were obtained from the splenocytes of mice with CAC with or without MyD88 inhibitor treatment. For evaluation of MDSC suppressive activity, 2 × 10^5^ CD11b^+^Gr-1^+^ MDSCs and 2 × 10^5^ CD4^+^ splenic T cells from naive BalB/c mice were cultured in flat-bottom 96-well plates in complete RPMI medium. The proliferation assay of CD4^+^ T cells was performed using a carboxy-fluorescein diacetate succinimidyl ester (CFSE, Invitrogen, USA) assay, according to the manufacturer’s protocol. T cells were stimulated by the addition of anti-CD3/anti-CD28-antibody-coated microbeads (Invitrogen, USA) at a bead:cell ratio of 1:75 for 72 h.

### Statistical analysis

The data are expressed as the mean ± standard deviation (SD). Comparisons between groups were analyzed using Student’s t test or log rank test. Individual differences versus various controls were assessed using one-way ANOVA. All statistical analyses were performed using the SPSS software package (version 17.0; IBM Corp, Armonk, NY, USA). Statistical significance was indicated by a *P* value < 0.05.

## Results

### MDSC expansion during chronic colitis and CAC in mice

**Fig. 1:**
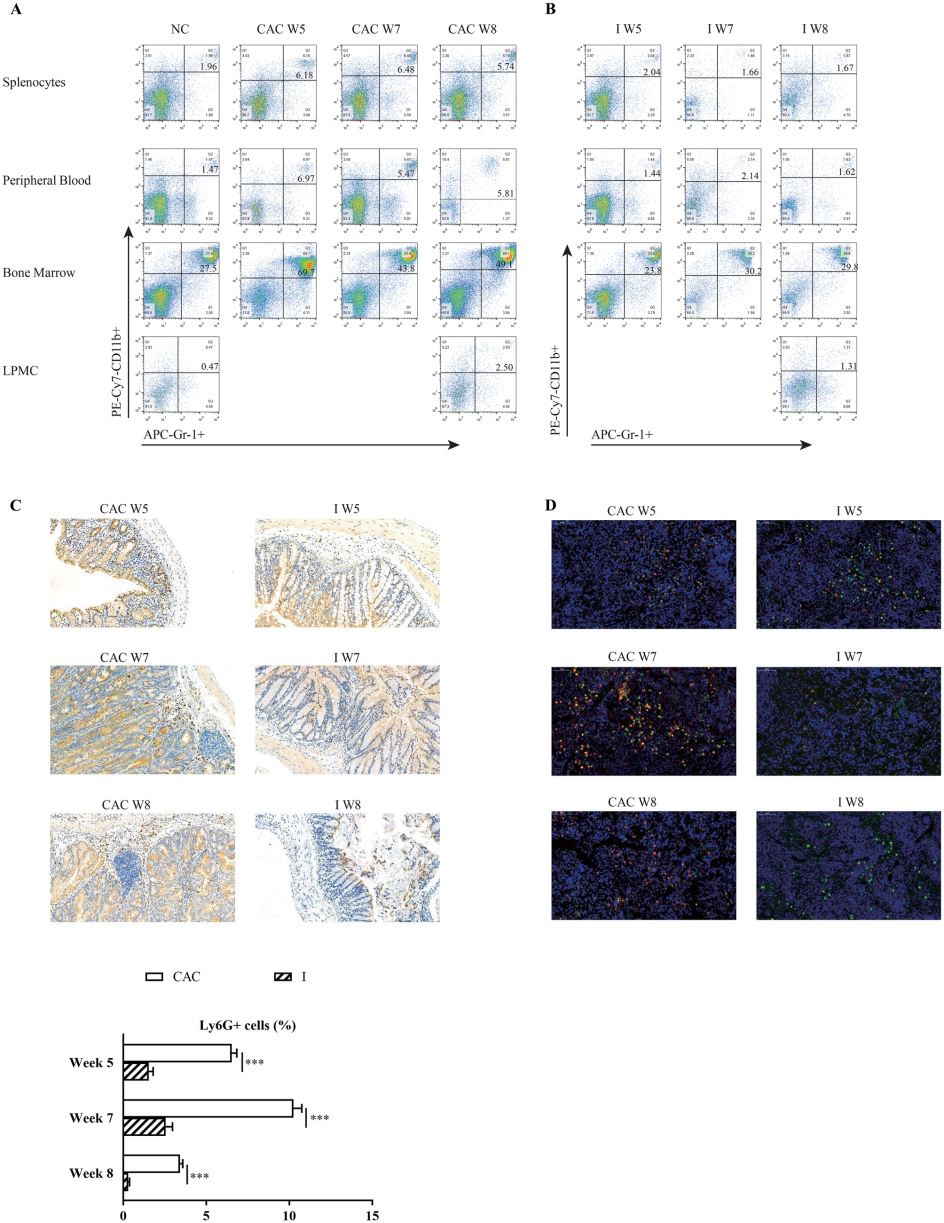
(**A, B**) Population of CD11b^+^Gr-1^+^ MDSCs in the spleen, PB, BM, and LP by flow cytometry five, seven and eight weeks post-induction (W5, W7 and W8) in the CAC group and the normal control group (NC, A) and in the MyD88 inhibitor-treated group (I, B). N = 5 per group. C&D) IHC staining for Ly6G^+^ cells of colon sections (**C**) and IF staining for CD11b^+^ (green) Ly6G^+^ (red) cells of spleen sections (**D**) in the CAC group and the I group five, seven and eight weeks post-induction (W5, W7 and W8). Scar bar = 50 μM. Histogram at the bottom (**C**) shows the percentage of positive Ly6G^+^ cells in total cells. N = 4 per group. Data are expressed as the mean ± SD of each group. ***P < 0.001

Our previous report proved that mice with AOM/DSS-induced CAC developed multiple tumor lesions in the colon over a 10-week period. Severe colitis and an increased frequency of tumor growth were observed in these colons [33]. Furthermore, we investigated the presence of different populations of myeloid cells. These mice had a significantly higher proportion (1.6–4.7-fold that of the control) of CD11b^+^Gr-1^+^MDSCs in the spleen, PB, and BM five weeks post-induction and a higher proportion (> fivefold that of the control) in the LP eight weeks post-induction (Fig. 1A). These results indicated that MDSC accumulation in the CAC model aggravated both colitis and tumor development.

### MyD88 inhibitor (TJ-M2010-5) treatment reduces the accumulation of MDSCs in mice with AOM/DSS-induced CAC in the absence of MyD88 signaling through

We previously showed that MyD88 signaling blockade with MyD88 inhibitor treatment in mice with AOM/DSS-induced CAC leads to reduced incidence of colitis and complete suppression of CAC development. We assessed the effects of the MyD88 inhibitor on MDSC accumulation during CAC development. Our flow cytometry data showed that the populations of CD11b^+^Gr-1^+^ MDSCs in the LP, spleen, PB, and BM in mice with CAC treated with MyD88 inhibitor were significantly decreased to 40% (W8); 33% (W5) and 29% (W8); 21% (W5) and 28% (W8); 34% (W5) and 61% (W8), respectively, compared to those in mice with CAC (Fig. 1B). Consistent with these data, we noted that Ly6G^+^ cells were significantly reduced by approximately 75%-90% in the colon (Fig. 1C), and fewer CD11b^+^Ly6G^+^ cells in the spleen (Fig. 1D) were noted five weeks post-induction, compared with those in mice with CAC not receiving MyD88 inhibitor treatment, as determined by IHC or IF. Thus, blocking MyD88 signaling by MyD88 inhibitor treatment resulted in the reduced accumulation of MDSCs during CAC development.

### MyD88 signaling blockade reduces the expression of factors associated with MDSC accumulation in mice with AOM/DSS-induced CAC

**Fig. 2:**
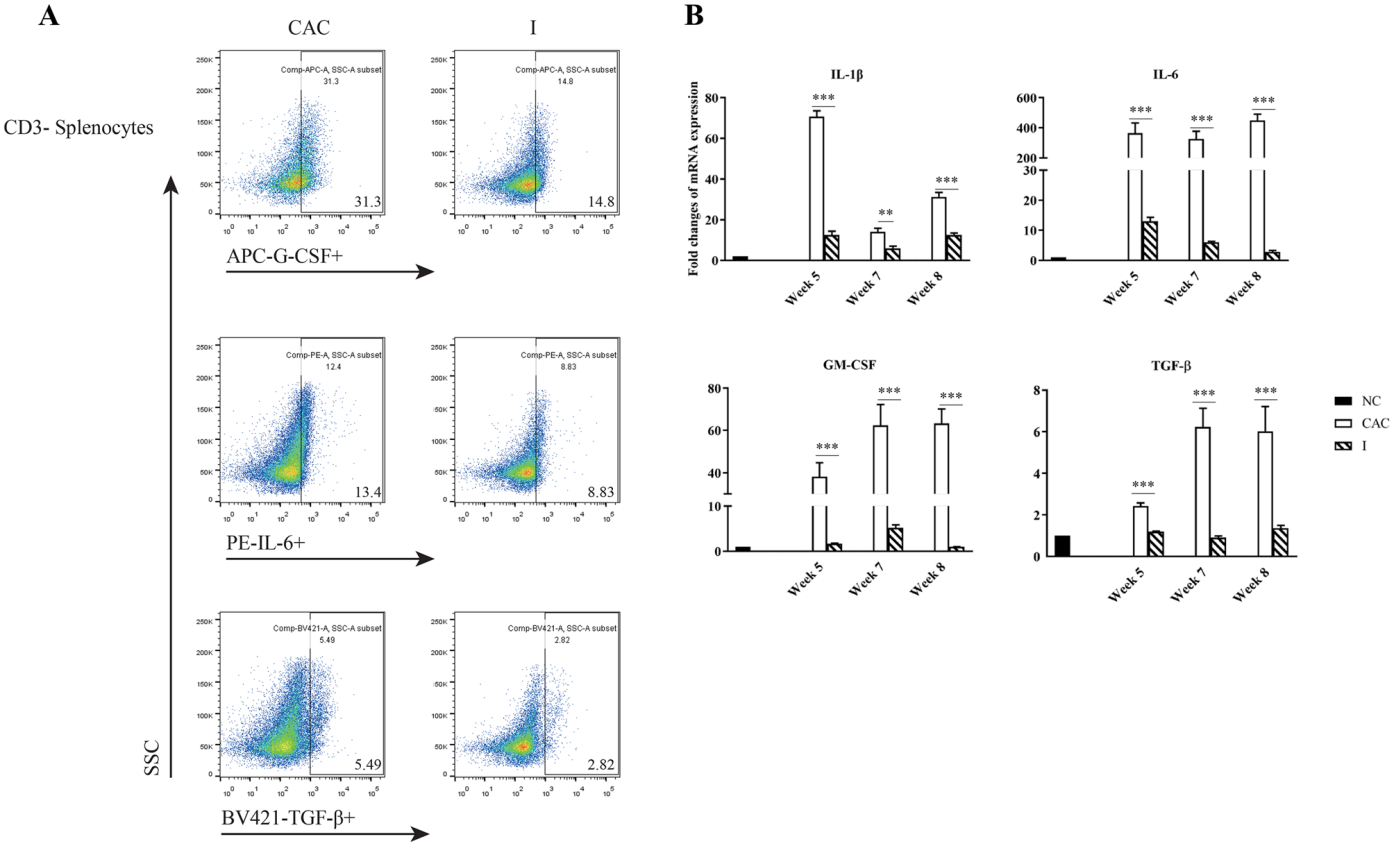
MyD88 inhibitor treatment reduced the expression of factors associated with MDSC accumulation in mice with CAC. **A**) Population of G-CSF, IL-6 and TGF-β-producing CD3^−^ splenocytes by flow cytometry eight weeks post-induction. **B**) Relative levels of GM-CSF, IL-1β, IL-6 and TGF-β mRNA transcripts in mouse colons eight weeks post-induction examined by RT-qPCR. Data are expressed as the mean ± SD of each group. All data of RT-qPCR were normalized to that in normal control mice. **P < 0.01; ***P < 0.001. N = 5 per group. Groups: the normal control (NC), CAC and MyD88 inhibitor-treated (I) groups

A MDSC originates from a common myeloid progenitor. MDSC development is supported by several growth factors (GM-CSF and G-CSF) and other factors, including IL-1β, IL-6, IL-11, IFN-γ, and tumor-derived exosomes, through the action of TLR2 and TGF-β, which have been implicated in the activation of signal transducer and activator of transcription (STAT)1, STAT3, and nuclear factor kappa-B (NF-κB) and the down-regulation of IFN regulatory factor 8 (IRF8) expression in MDSCs; these factors are critical for MDSC expansion [34]. Under pathological conditions of tumors or chronic inflammation, myeloid cells are unable to effectively differentiate into mature myeloid cells, caused by the persistent stimulation of the aforementioned factors, and the conversion of immature myeloid cells into MDSCs, which thereby lead to potent immune-suppressive effects [16]. Thus, we tested whether the levels of the factors supporting MDSC expansion in the absence of MyD88 signaling are decreased in mice with CAC after MyD88 inhibitor treatment. As shown in Fig. 2A, treatment with the MyD88 inhibitor significantly decreased the levels of G-CSF (to 47.3% of the CAC group), IL-6 (to 69.0% of the CAC group), and TGF-β (to 50.2% of the CAC group)-producing CD3^−^ splenocytes in mice with AOM/DSS-induced CAC eight weeks post-induction, findings that were consistent with our previous findings. Furthermore, a similar pattern in GM-CSF, IL-1β, IL-6, and TGF-β mRNA expression was detected in mouse colon tissue (Fig. 2B). These results indicate that MyD88 signaling blockade may disrupt tumor/inflammation-derived factor secretion associated with MDSC accumulation.

### Impact of MyD88 signaling blockade on the expression of molecules involved in MDSC-mediated immune suppression in mice with AOM/DSS-induced CAC

**Fig. 3:**
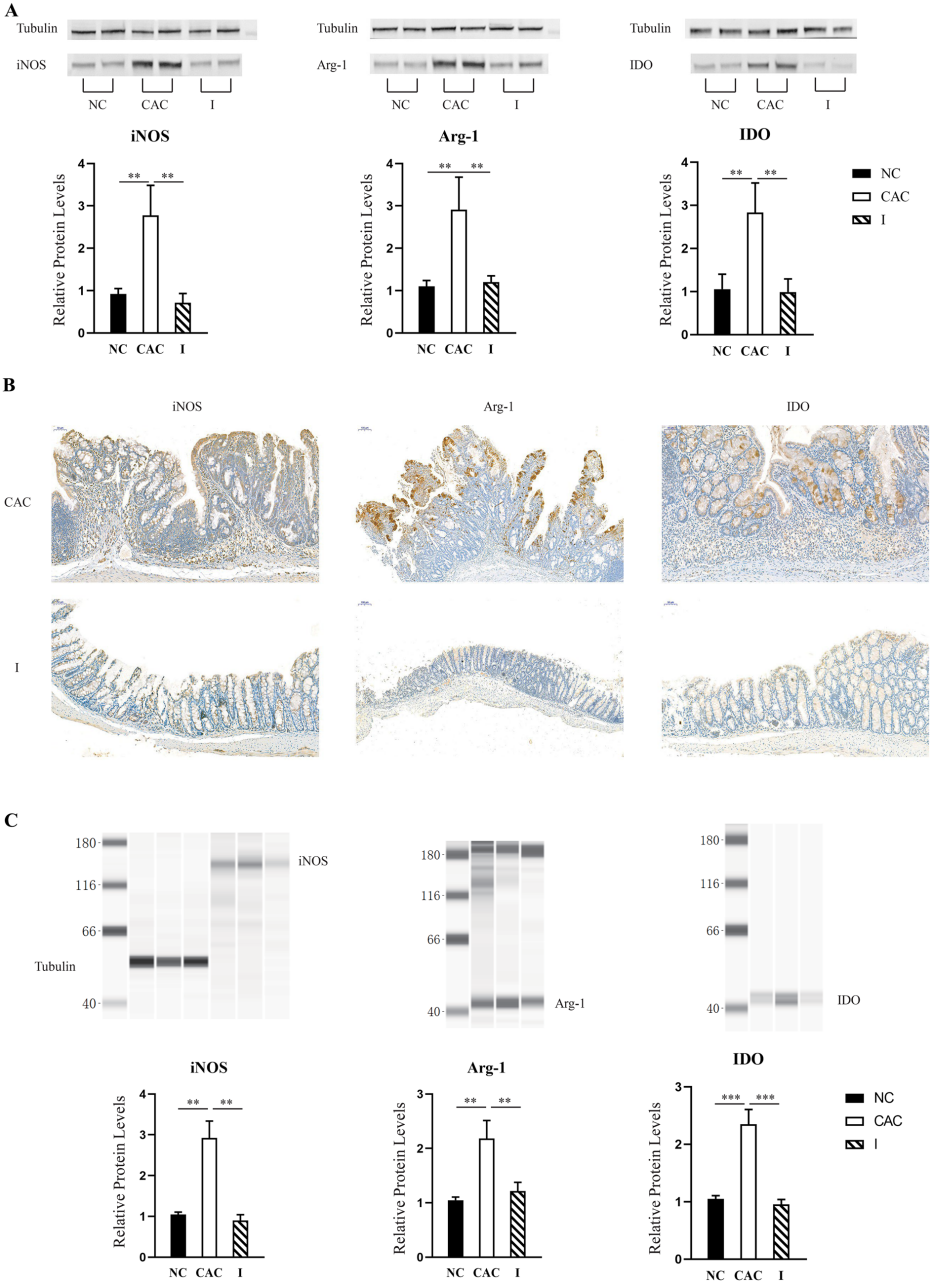
Impact of MyD88 inhibitor on the expression of molecules involved in MDSC-mediated immune suppression in mice with CAC. **A**) Protein extracts from colon tissue were analyzed by Western blotting for iNOS, Arg-1 and IDO. α-tubulin was used as a control. Data are representative images or expressed as the mean ± SD of each group. Densitometry analysis was performed with Image J software for quantification. **P < 0.01. **B**) Representative IHC stains for iNOS, Arg-1 and IDO in colonic samples seven weeks post-induction. Scale bar = 100 μM. N = 4 per group. Groups: the normal control (NC), CAC and MyD88 inhibitor-treated (I) groups. **C**) Protein extracts from cell lysates of sorted CD11b^+^Gr-1^+^ splenocytes were analyzed by WES for iNOS, Arg-1 and IDO. α-tubulin was used as a control. Data are representative images or expressed as the mean ± SD of each group. Quantitative analysis was performed using Compass software (Protein Simple). **P < 0.01; ***P < 0.001

Through a number of mechanisms, MDSCs mediate immune suppression through the upregulation of iNOS expression, leading to reactive oxygen species (ROS) production, upregulating Arg-1 expression by TGF-β and IL-10, promoting the immunosuppressive molecule IDO expression, and directly affecting CD4^+^ and CD8^+^ T cells [35]. To clarify the impact of MyD88 signaling blockade on MDSCs, we detected the expression of iNOS, Arg-1 and IDO in the colon tissue of mice with AOM/DSS-induced CAC after MyD88 inhibitor treatment. As shown in Fig. 3A, the expression of iNOS, Arg-1 and IDO was significantly reduced compared to that in mice with CAC, as assessed by Western blotting; a decrease of 55–75% was observed seven weeks post-induction. As presented in Fig. 3B, IHC staining revealed fewer iNOS-, Arg-1- and IDO- positive cells infiltrating into the LP of the colon, compared to mice with CAC. We detected the expression of these proteins in CD11b^+^Gr-1^+^ MDSCs obtained from the spleens of the mice. The expression of iNOS, Arg-1 and IDO was significantly decreased in mice treated with the MyD88 inhibitor compared to mice with CAC, as determined by WES; a decrease of 45–70% was seen seven weeks post-induction (Fig. 3C). Thus, MyD88 signaling blockade not only reduced the population of MDSCs, but also downregulated the expression of molecules involved in the suppressive capacity of MDSCs in mice with CAC.

### MyD88 signaling blockade suppressed the differentiation of myeloid cells into MDSCs in vitro

**Fig. 4:**
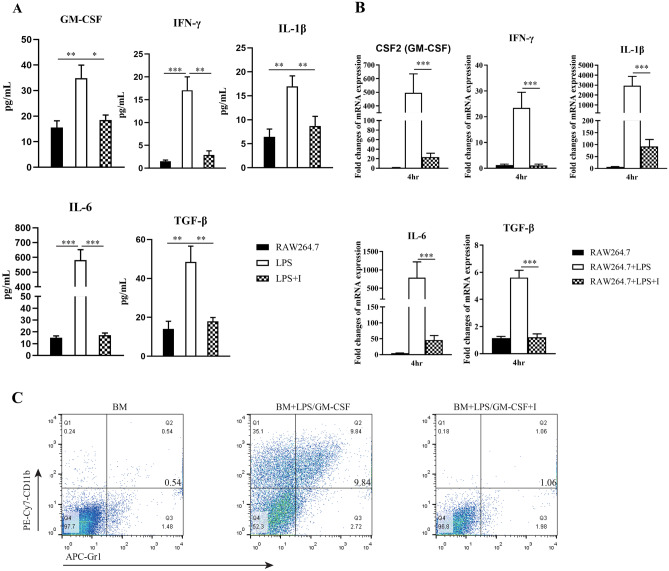
MyD88 inhibitor administration suppressed the differentiation of myeloid cells into MDSCs in vitro. Supernatant concentrations detected by ELISA (**A**) and relative levels of mRNA transcripts detected by RT-qPCR (**B**) of GM-CSF, IFN-γ, IL-1β, IL-6 and TGF-β1 in RAW 264.7 cells are shown. Data are expressed as the mean ± SD of each group from three independent experiments. Data from the RT-qPCR were normalized to unstimulated cells. *P < 0.05; **P < 0.01; ***P < 0.001. **C**) BM cells were cultured in the present of GM-CSF and LPS for eight days and a phenotypic analysis on induced CD11b^+^Gr-1^+^ MDSCs was performed by flow cytometry. Groups: the unstimulated, LPS or LPS/GM-CSF-stimulated and MyD88 inhibitor-treated (I) cells groups

Macrophages are important sources of cytokines involved in MDSC expansion. To determine whether MyD88 signaling was critical for the generation of MDSCs in vitro, the cytokine secretion profile of RAW 264.7 cells in the presence of LPS with or without MyD88 inhibitor treatment was determined. As shown in Fig. 4A, significantly elevated GM-CSF (*p* = 0.004), IFN-γ (*p* = 0.0008), IL-1β (*p* = 0.003), IL-6 (*p* = 0.0002), and TGF-β1 (*p* = 0.003) levels were confirmed in RAW 264.7 cells stimulated with LPS compared to those in unstimulated cells. However, after treatment with the MyD88 inhibitor, the supernatant concentration of these cytokines was significantly reduced to levels nearly identical to the levels of unstimulated cells. A similar pattern in the mRNA expression of these cytokines was also observed (Fig. 4B). MyD88 inhibitor treatment greatly reduced GM-CSF, IFN-γ, IL-1β, IL-6, and TGF-β1 mRNA expression in RAW 264.7 cells in the presence of LPS. In addition, in the presence of GM-CSF and LPS, CD11b^+^Gr-1^+^ MDSCs were generated from CD11b^−^ BM progenitor cells of BalB/c mice in vitro, with an increased population of 9.83% vs. that of 0.54% at baseline; this induction was diminished (1.06%) by MyD88 inhibitor treatment (Fig. 4C). There results suggest that the blockade initiated by the MyD88 inhibitor not only inhibited MDSC expansion by decreasing the secretion of related cytokines but also direct suppressed MDSC differentiation.

### Loss of MDSC suppression on CD4^+^ T cell proliferation after MyD88 inhibitor administration

**Fig. 5:**
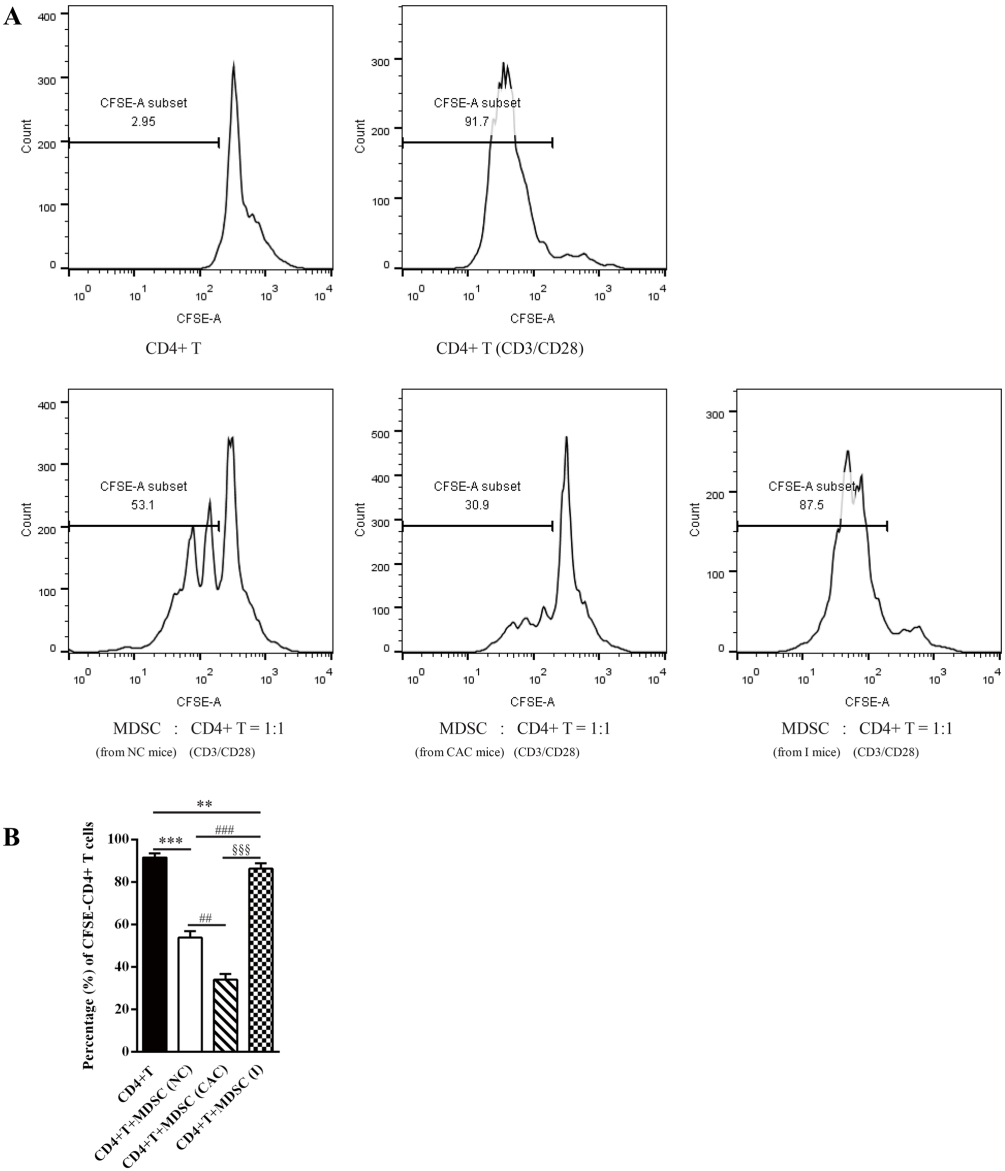
The suppressive capacity of CD11b^+^Gr-1^+^ MDSCs on the proliferation of activated CD4^+^ T cells was reserved by MyD88 inhibitor administration. MDSCs derived from the normal control, CAC and MyD88 inhibitor-treated (I) mice were co-cultured with CFSE-labeled autologous CD4^+^ T cells in proportion (1:1) in the present of anti-CD3/anti-CD28-antibody-coated microbeads. **A**) CFSE-labeled T cells were analyzed by flow cytometry for proliferation. **B**) Percentage of CFSE-labeled CD4+ T cells were stimulated by anti-CD3/anti-CD28-antibodies. Data were from three independent experiments. Values are means ± SD. ***P < 0.001, **P < 0.01 vs. stimulated CD4+ T cells; ###P <0.001, ##P < 0.01 vs. stimulated CD4+ T cells co-cultured with MDSCs from NC mice; §§§P <0.001 vs. stimulated CD4+ T cells co-cultured with MDSCs from CAC mice

Next, we examined the effect of MyD88 inhibitor therapy on the suppressive function of MDSCs in mice with AOM/DSS-induced CAC. As shown in Fig. 5, the proliferation of CD4^+^ T cells activated by CD3/CD28 antibodies declined from 91.7% to 53.1% when MDSCs were cultured in normal control mice in vitro. The proliferation rate of CD4^+^ T cells further decreased to 30.9% in MDSCs of mice with CAC. MDSCs, especially from tumor-bearing hosts, effectively suppressed CD4^+^ T cell proliferation. However, MDSCs derived from the spleen of MyD88 inhibitor-treated mice showed a significantly less suppressive effect on the proliferation of activated CD4^+^ T cells, with an attenuated proliferation rate of 87.5%. Thus, these results clearly suggested that MyD88 signaling may be crucial to the suppressive capacity of MDSCs.

## Discussion

Colorectal cancer is the third most common malignancy in humans; furthermore, it is the second leading cause of cancer-related death in most countries. Several studies have confirmed that colitis is one of the primary drivers of colon tumorigenesis [36]. During the development of inflammation and carcinogenesis in the colon, the innate immune system plays a critical role in protecting against CAC by triggering antimicrobial responses, maintaining intestinal homeostasis, and inducing healing; on the other hand, it promotes chronic inflammation in the tumor microenvironment and the evasion of antitumor effectors during immune responses [37]. Thus, understanding the dual role of the innate immune system in cancer immunology is vital to designing effective therapeutic strategies against CAC.

Chronic inflammation and tumors may actively recruit and condition a complex network of cells belonging to the innate immune system, such as tumor-associated macrophages (TAMs), NKTs, and MDSCs. Among these cells, MDSCs are a heterogeneous population of immune cells that can impair the antitumor response both locally, in the tumor site, and systemically, in lymphoid organs. A growing number of studies have demonstrated that targeting MDSCs improves the effect of anticancer therapies in breast, colorectal, prostate, hepatocellular and melanoma cancer [38–42]. In this study, we also proved the contribution of MDSCs to the development of CAC driven by AOM/DSS administration in mice. We observed the accumulation of cells with a typical MDSC phenotype (CD11b^+^Gr-1^+^) in the spleen, PB, and BM of mice at five weeks post-induction, when colitis was severe, and in the LP at eight weeks post-induction, when the colons were full of neoplastic lesions. These data indicated that MDSC accumulation in this model was a result of both chronic colitis and tumor formation. In addition, MDSC accumulation due to inflammation occurred earlier in systemic lymphoid organs than in the local tumor site as a consequence of cancer development.

Since MDSCs are important to inflammation and thus to carcinogenesis, it is essential to understand the molecular mechanisms of expanding and activating MDSCs because they may be potential targets for cancer immunotherapy. It has been proven that signaling through an Myd88 adaptor molecule is critical for the suppressive function of MDSCs. Blocking MyD88 signaling through the transgenic method has succeeded in inhibiting tumor growth in lung and ovarian in mouse model because it contributes to MDSC suppression [26, 43]. Although inhibition of MyD88 signaling in MDSCs is proving to be a promising treatment for cancer, effective administration of these agents has not yet been accomplished. Our previous study has proven that the novel synthetic MyD88 inhibitor (TJ-M2010-5) is an effective inhibitor of MyD88 homodimerization, and its administration successfully prevents the development of CAC in mice [33]. In the present study, we showed that the accumulation and differentiation of MDSCs were dependent on both MyD88 signaling activation and TJ-M2010-5 inhibitor administration. These results are shown in Figs. 1B and 4C and they reveal a decline in innate immune cells (e.g. macrophages) or cancer-derived factors both in vivo (Fig. 2) and in vitro (Fig. 4A, B). We have proven that MDSCs are generated and that they proliferate and migrate in response to these cytokines and chemokines; furthermore, MyD88 signaling is essential for inflammation and tumor cell secretion [44]. Thus, TJ-M2020-5, a MyD88 inhibitor, has the potential to be an available clinical agent that acts on MDSCs to prevent CAC development.

Immunosuppressive MDSCs have been implicated in tumor immune evasion through the following mechanisms [45–47]: (i) stimulating the production of ROS, which decreases T cell receptor functionality through NADPH oxidase and iNOS; (ii) producing high Arg-1 levels, which deplete T cells of L-arginine and induce cell cycle arrest; and (iii) synthesizing IDO to protect tumors from attack by specific tumor T cells by inducing tolerance through tryptophan catabolism to inhibit T cell proliferation and induce Treg cells, among others. In this study, we showed that the immunosuppressive functions of MDSCs depend on MyD88 signaling activation. We found that MyD88 inhibitor administration reduced iNOS, Arg-1 and IDO expression to weaken tumor-immune evasion mediated by MDSCs (Fig. 3). Thus, the immunosuppressive capabilities of MDSCs also depend on MyD88 signaling activation. On the other hand, it has been demonstrated that intratumor MDSCs mainly hamper CD8^+^ T cell activation, which t suppresses tumor cell escape from the immune system [48, 49]; however, the ability of MDSCs to elicit CD4^+^ T cell tolerance is still debated and unclear. Our results (Fig. 5) showed that MDSCs derived from mice with CAC showed a significant ability to inhibit CD4^+^ T cell proliferation in vitro and that a MyD88 signaling blockade caused by the MyD88 inhibitor reduced the inhibitory effect of MDSCs on CD4^+^ T cell proliferation. Another study has also proved that MDSCs from mice with chronic infection suppress OVA-specific CD4^+^ T cell proliferation via a nitric oxide-dependent mechanism [50]. These findings indicate that CAC development induces MDSCs that suppress CD4^+^ T cell proliferation and promote inflammation associated with carcinogenesis.

Indeed, the regulation of MDSC immunosuppressive functions plays a critical role in successful immunotherapy against cancer; moreover, Myd88 signaling is involved in the regulation of the immunosuppressive functions of MDSCs. Our novel MyD88 inhibitor, TJ-M2010-5, is a new and effective agent to modulate MyD88 signaling, which overcomes MDSC suppressive functions, making it a successful antitumor therapy.

## Data Availability

All data generated or analyzed during this study are included in this article.

## References

[1] Talmadge JE, Gabrilovich DI (2013). History of myeloid-derived suppressor cells. Nat Rev Cancer.

[2] Kerkar SP, Restifo NP (2012). Cellular constituents of immune escape within the tumor microenvironment. Can Res.

[3] Kirkwood JM, Tarhini AA, Panelli MC, Moschos SJ, Zarour HM, Butterfield LH et al (2008) Next generation of immunotherapy for melanoma. J Clin Oncol: Official J Am Soc Clin Oncol 26:3445–55. 10.1200/JCO.2007.14.6423 10.1200/JCO.2007.14.6423 18612161

[4] Motz GT, Coukos G (2013). Deciphering and reversing tumor immune suppression. Immunity.

[5] Filipazzi P, Huber V, Rivoltini L (2012). Phenotype, function and clinical implications of myeloid-derived suppressor cells in cancer patients. Cancer immunology, immunotherapy : CII.

[6] Gabrilovich DI, Nagaraj S (2009). Myeloid-derived suppressor cells as regulators of the immune system. Nat Rev Immunol.

[7] Ostrand-Rosenberg S (2010). Myeloid-derived suppressor cells: more mechanisms for inhibiting antitumor immunity. Cancer immunology, immunotherapy : CII.

[8] Khaled YS, Ammori BJ, Elkord E (2013). Myeloid-derived suppressor cells in cancer: recent progress and prospects. Immunol Cell Biol.

[9] Alizadeh D, Larmonier N (2014). Chemotherapeutic targeting of cancer-induced immunosuppressive cells. Can Res.

[10] Umansky V, Sevko A (2012). Melanoma-induced immunosuppression and its neutralization. Semin Cancer Biol.

[11] Dolcetti L, Peranzoni E, Ugel S, Marigo I, Fernandez Gomez A, Mesa C (2010). Hierarchy of immunosuppressive strength among myeloid-derived suppressor cell subsets is determined by GM-CSF. Eur J Immunol.

[12] Tu S, Bhagat G, Cui G, Takaishi S, Kurt-Jones EA, Rickman B (2008). Overexpression of interleukin-1beta induces gastric inflammation and cancer and mobilizes myeloid-derived suppressor cells in mice. Cancer Cell.

[13] Bunt SK, Yang L, Sinha P, Clements VK, Leips J, Ostrand-Rosenberg S (2007). Reduced inflammation in the tumor microenvironment delays the accumulation of myeloid-derived suppressor cells and limits tumor progression. Can Res.

[14] Melani C, Sangaletti S, Barazzetta FM, Werb Z, Colombo MP (2007). Amino-biphosphonate-mediated MMP-9 inhibition breaks the tumor-bone marrow axis responsible for myeloid-derived suppressor cell expansion and macrophage infiltration in tumor stroma. Can Res.

[15] Takaku S, Terabe M, Ambrosino E, Peng J, Lonning S, McPherson JM (2010). Blockade of TGF-beta enhances tumor vaccine efficacy mediated by CD8(+) T cells. Int J Cancer.

[16] Kumar V, Patel S, Tcyganov E, Gabrilovich DI (2016). The Nature of Myeloid-Derived Suppressor Cells in the Tumor Microenvironment. Trends Immunol.

[17] Li H, Han Y, Guo Q, Zhang M, Cao X (2009). Cancer-expanded myeloid-derived suppressor cells induce anergy of NK cells through membrane-bound TGF-beta 1. J Immunol.

[18] Sinha P, Clements VK, Bunt SK, Albelda SM, Ostrand-Rosenberg S (2007). Cross-talk between myeloid-derived suppressor cells and macrophages subverts tumor immunity toward a type 2 response. J Immunol.

[19] Egelston C, Kurko J, Besenyei T, Tryniszewska B, Rauch TA, Glant TT (2012). Suppression of dendritic cell maturation and T cell proliferation by synovial fluid myeloid cells from mice with autoimmune arthritis. Arthritis Rheum.

[20] Sumpter TL, Falo LD (2014). "Toll"-erance in the skin. Immunity.

[21] Skabytska Y, Wolbing F, Gunther C, Koberle M, Kaesler S, Chen KM (2014). Cutaneous innate immune sensing of Toll-like receptor 2–6 ligands suppresses T cell immunity by inducing myeloid-derived suppressor cells. Immunity.

[22] Arora M, Poe SL, Oriss TB, Krishnamoorthy N, Yarlagadda M, Wenzel SE (2010). TLR4/MyD88-induced CD11b+Gr-1 int F4/80+ non-migratory myeloid cells suppress Th2 effector function in the lung. Mucosal Immunol.

[23] Zoglmeier C, Bauer H, Noerenberg D, Wedekind G, Bittner P, Sandholzer N (2011). CpG blocks immunosuppression by myeloid-derived suppressor cells in tumor-bearing mice. Clinical cancer research : an official journal of the American Association for Cancer Research.

[24] Siednienko J, Gajanayake T, Fitzgerald KA, Moynagh P, Miggin SM (2011). Absence of MyD88 results in enhanced TLR3-dependent phosphorylation of IRF3 and increased IFN-beta and RANTES production. J Immunol.

[25] Ray A, Chakraborty K, Ray P (2013). Immunosuppressive MDSCs induced by TLR signaling during infection and role in resolution of inflammation. Front Cell Infect Microbiol.

[26] Hong EH, Chang SY, Lee BR, Kim YS, Lee JM, Kang CY (2013). Blockade of Myd88 signaling induces antitumor effects by skewing the immunosuppressive function of myeloid-derived suppressor cells. Int J Cancer.

[27] Liu J, Zhang X, Wang H, Zhang M, Peng Y, Li M (2019). Implication of myeloid differentiation factor 88 inhibitor TJ-M2010-5 for therapeutic intervention of hepatocellular carcinoma. Hepatology research : the official journal of the Japan Society of Hepatology.

[28] Ding Z, Du D, Yang Y, Yang M, Miao Y, Zou Z (2019). Short-term use of MyD88 inhibitor TJ-M2010-5 prevents d-galactosamine/lipopolysaccharide-induced acute liver injury in mice. Int Immunopharmacol.

[29] Yang M, Chen G, Zhang X, Ding Z, Miao Y, Yang Y (2019). A novel MyD88 inhibitor attenuates allograft rejection after heterotopic tracheal transplantation in mice. Transpl Immunol.

[30] Li C, Zhang LM, Zhang X, Huang X, Liu Y, Li MQ (2017). Short-term Pharmacological Inhibition of MyD88 Homodimerization by a Novel Inhibitor Promotes Robust Allograft Tolerance in Mouse Cardiac and Skin Transplantation. Transplantation.

[31] Xing S, Zhang X, Huang X, Xie L, Jiang F, Zhou P (2019). Modulating the conformation of the TIR domain by a neoteric MyD88 inhibitor leads to the separation of GVHD from GVT. Leuk Lymphoma.

[32] Miao Y, Ding Z, Zou Z, Yang Y, Yang M, Zhang X (2020). Inhibition of MyD88 by a novel inhibitor reverses two-thirds of the infarct area in myocardial ischemia and reperfusion injury. American journal of translational research.

[33] Xie L, Jiang FC, Zhang LM, He WT, Liu JH, Li MQ et al (2016) Targeting of MyD88 Homodimerization by Novel Synthetic Inhibitor TJ-M2010-5 in Preventing Colitis-Associated Colorectal Cancer. J Natl Cancer Inst 108.10.1093/jnci/djv364 10.1093/jnci/djv364 26712311

[34] Condamine T, Mastio J, Gabrilovich DI (2015). Transcriptional regulation of myeloid-derived suppressor cells. J Leukoc Biol.

[35] Condamine T, Gabrilovich DI (2011). Molecular mechanisms regulating myeloid-derived suppressor cell differentiation and function. Trends Immunol.

[36] Saleh M, Trinchieri G (2011). Innate immune mechanisms of colitis and colitis-associated colorectal cancer. Nat Rev Immunol.

[37] Berraondo P, Minute L, Ajona D, Corrales L, Melero I, Pio R (2016). Innate immune mediators in cancer: between defense and resistance. Immunol Rev.

[38] De Cicco P, Ercolano G, Ianaro A (2020). The New Era of Cancer Immunotherapy: Targeting Myeloid-Derived Suppressor Cells to Overcome Immune Evasion. Front Immunol.

[39] Katoh H, Wang D, Daikoku T, Sun H, Dey SK, Dubois RN (2013). CXCR2-expressing myeloid-derived suppressor cells are essential to promote colitis-associated tumorigenesis. Cancer Cell.

[40] Chun E, Lavoie S, Michaud M, Gallini CA, Kim J, Soucy G (2015). CCL2 Promotes Colorectal Carcinogenesis by Enhancing Polymorphonuclear Myeloid-Derived Suppressor Cell Population and Function. Cell Rep.

[41] Holmgaard RB, Zamarin D, Lesokhin A, Merghoub T, Wolchok JD (2016). Targeting myeloid-derived suppressor cells with colony stimulating factor-1 receptor blockade can reverse immune resistance to immunotherapy in indoleamine 2,3-dioxygenase-expressing tumors. EBioMedicine.

[42] Mao Y, Eissler N, Blanc KL, Johnsen JI, Kogner P, Kiessling R (2016). Targeting Suppressive Myeloid Cells Potentiates Checkpoint Inhibitors to Control Spontaneous Neuroblastoma. Clinical cancer research : an official journal of the American Association for Cancer Research.

[43] Baert T, Vankerckhoven A, Riva M, Van Hoylandt A, Thirion G, Holger G (2019). Myeloid Derived Suppressor Cells: Key Drivers of Immunosuppression in Ovarian Cancer. Front Immunol.

[44] Di Mitri D, Toso A, Alimonti A (2015). Molecular Pathways: Targeting Tumor-Infiltrating Myeloid-Derived Suppressor Cells for Cancer Therapy. Clinical cancer research : an official journal of the American Association for Cancer Research.

[45] Kulsantiwong P, Pudla M, Srisaowakarn C, Boondit J, Utaisincharoen P (2017). Pam2CSK4 and Pam3CSK4 induce iNOS expression via TBK1 and MyD88 molecules in mouse macrophage cell line RAW264.7. Inflammation research : official journal of the European Histamine Research Society [et al].

[46] Liu JT, Wu SX, Zhang H, Kuang F (2018). Inhibition of MyD88 Signaling Skews Microglia/Macrophage Polarization and Attenuates Neuronal Apoptosis in the Hippocampus After Status Epilepticus in Mice. Neurotherapeutics : the journal of the American Society for Experimental NeuroTherapeutics.

[47] Liu H, Zhang G, Huang J, Ma S, Mi K, Cheng J (2016). Atractylenolide I modulates ovarian cancer cell-mediated immunosuppression by blocking MD-2/TLR4 complex-mediated MyD88/NF-kappaB signaling in vitro. J Transl Med.

[48] Nagaraj S, Gupta K, Pisarev V, Kinarsky L, Sherman S, Kang L (2007). Altered recognition of antigen is a mechanism of CD8+ T cell tolerance in cancer. Nat Med.

[49] Schouppe E, Mommer C, Movahedi K, Laoui D, Morias Y, Gysemans C (2013). Tumor-induced myeloid-derived suppressor cell subsets exert either inhibitory or stimulatory effects on distinct CD8+ T-cell activation events. Eur J Immunol.

[50] Valanparambil RM, Tam M, Jardim A, Geary TG, Stevenson MM (2017). Primary Heligmosomoides polygyrus bakeri infection induces myeloid-derived suppressor cells that suppress CD4(+) Th2 responses and promote chronic infection. Mucosal Immunol.

